# Case Report: Life-Threatening Macrophage Activation Syndrome With Fulminant Myocarditis Successfully Rescued by High Dose Intravenous Anakinra

**DOI:** 10.3389/fped.2020.635080

**Published:** 2021-01-18

**Authors:** Alessandra Meneghel, Giorgia Martini, Angela Amigoni, Andrea Pettenazzo, Massimo Padalino, Francesco Zulian

**Affiliations:** ^1^Pediatric Rheumatology Unit, Department of Women's and Children's Health, University Hospital of Padua, Padua, Italy; ^2^Pediatric Intensive Care Unit, Department of Women's and Children's Health, University Hospital of Padua, Padua, Italy; ^3^Pediatric and Congenital Cardiac Surgery, Department of Cardiac, Thoracic and Vascular Sciences, University of Padua, Padua, Italy

**Keywords:** macrophage activation syndrome, anakinra, systemic juvenile idiopathic arthritis, myocarditis, ECMO

## Abstract

Macrophage activation syndrome (MAS) is a rare, potentially life-threatening, condition triggered by infections or flares in rheumatologic and neoplastic diseases. The mainstay of treatment includes high dose corticosteroids, intravenous immunoglobulins and immunosuppressive drugs although, more recently, a more targeted approach, based on the use of selective cytokines inhibitors, has been reported. We present the case of a two-year-old boy with 1-month history of high degree fever associated with limping gait, cervical lymphadenopathy and skin rash. Laboratory tests showed elevation of inflammatory markers and ferritin. By exclusion criteria, systemic onset Juvenile Idiopathic Arthritis (sJIA) was diagnosed and steroid therapy started. A couple of weeks later, fever relapsed and laboratory tests were consistent with MAS. He was promptly treated with high doses intravenous methylprednisolone pulses and oral cyclosporin A. One day later, he developed an acute myocarditis and a systemic capillary leak syndrome needing intensive care. Intravenous Immunoglobulin and subcutaneous IL-1-antagonists Anakinra were added. On day 4, after an episode of cardiac arrest, venous-arterial extracorporeal membrane oxygenation (VA-ECMO) was started. Considering the severe refractory clinical picture, we tried high dose intravenous Anakinra (HDIV-ANA, 2 mg/Kg q6h). This treatment brought immediate benefit: serial echocardiography showed progressive resolution of myocarditis, VA-ECMO was gradually decreased and definitively weaned off in 6 days and MAS laboratory markers improved. Our case underscores the importance of an early aggressive treatment in refractory life-threatening sJIA-related MAS and adds evidence on safety and efficacy of HDIV-ANA particularly in acute myocarditis needing VA-ECMO support.

## Introduction

Macrophage activation syndrome (MAS) is a rare, potentially life-threatening complication of some rheumatologic diseases, such as systemic onset Juvenile Idiopathic Arthritis (sJIA), Kawasaki Disease (KD) and Systemic Lupus Erythematous (SLE), or a condition triggered by viral and or bacterial infections in predisposed individuals or associated with neoplastic disease (1). Early diagnosis is challenging as an appropriate therapy can significantly improve the outcome. The mainstay of treatment is the use of high dose corticosteroids (1, 2) but, in the last few years, the better understanding of the pathological pathways of the disease opened up new perspectives on the use of selective cytokines inhibitors (1). Recently Anakinra (ANA), a recombinant IL-1 receptor antagonist, has been successfully used for the treatment of sJIA-related MAS, pointing out the need for high doses in those refractory to conventional treatment (3–5). Acute myocarditis is a rare and potentially fatal complication of sJIA (6) and, given the rarity of this condition, experience on treatment is very limited. In adult-onset Still disease, single case reports suggested that ANA may play a role also for this complication (7–9). Taken together, these experiences suggest that ANA represents a potential effective treatment for MAS and myocarditis complicating sJIA.

We describe the case of a child with sJIA complicated by severe MAS, Systemic Capillary Leak Syndrome (SCLS) and acute myocarditis, leading to distributive and cardiogenic shock, cardiac arrest needing cardiopulmonary resuscitation and veno-arterial Extracorporeal Membrane Oxygenation (VA-ECMO), who was successfully treated with high dose intravenous ANA (HDIV-ANA).

## Case Report

A previously healthy two-year-old boy presented with 1 month history of fever associated with limping gait, cervical lymphadenopathy and evanescent skin rash. He was evaluated at a regional hospital where laboratory tests showed: WBC 25990/mm3 (N 18740/mm3); CRP 65 mg/L; ESR 68 mm/h; ferritin 1,259 ug/L; tryglicerides 1.5 mmol/L; AST 61 U/L, ALT 45 U/L. Echocardiography was normal. Bone marrow aspiration was negative for blasts. A short course of oral prednisone (1 mg/kg/day) was started with benefit on fever. However, upon steroid tapering, fever and limping reappeared. MRI showed synovial membrane hypertrophy and effusion in both hips. Therefore, according with ILAR criteria (10), sJIA was diagnosed and high dose pulse intravenous (IV) methylprednisolone (MPDN, 30 mg/Kg/day) was given for 2 days then followed by prednisone maintenance dose (2 mg/Kg/day). Two days later, due to a methicillin-resistant staphylococcus aureus cellulitis of the right hand, IV teicoplanin was started and the patient was referred to our Pediatric Rheumatology Unit.

On admission, physical examination revealed unremitting high-grade fever, erythematous skin rash on face and limbs and mild hepato-splenomegaly. No arthritis was detected.

**Figure 1:**
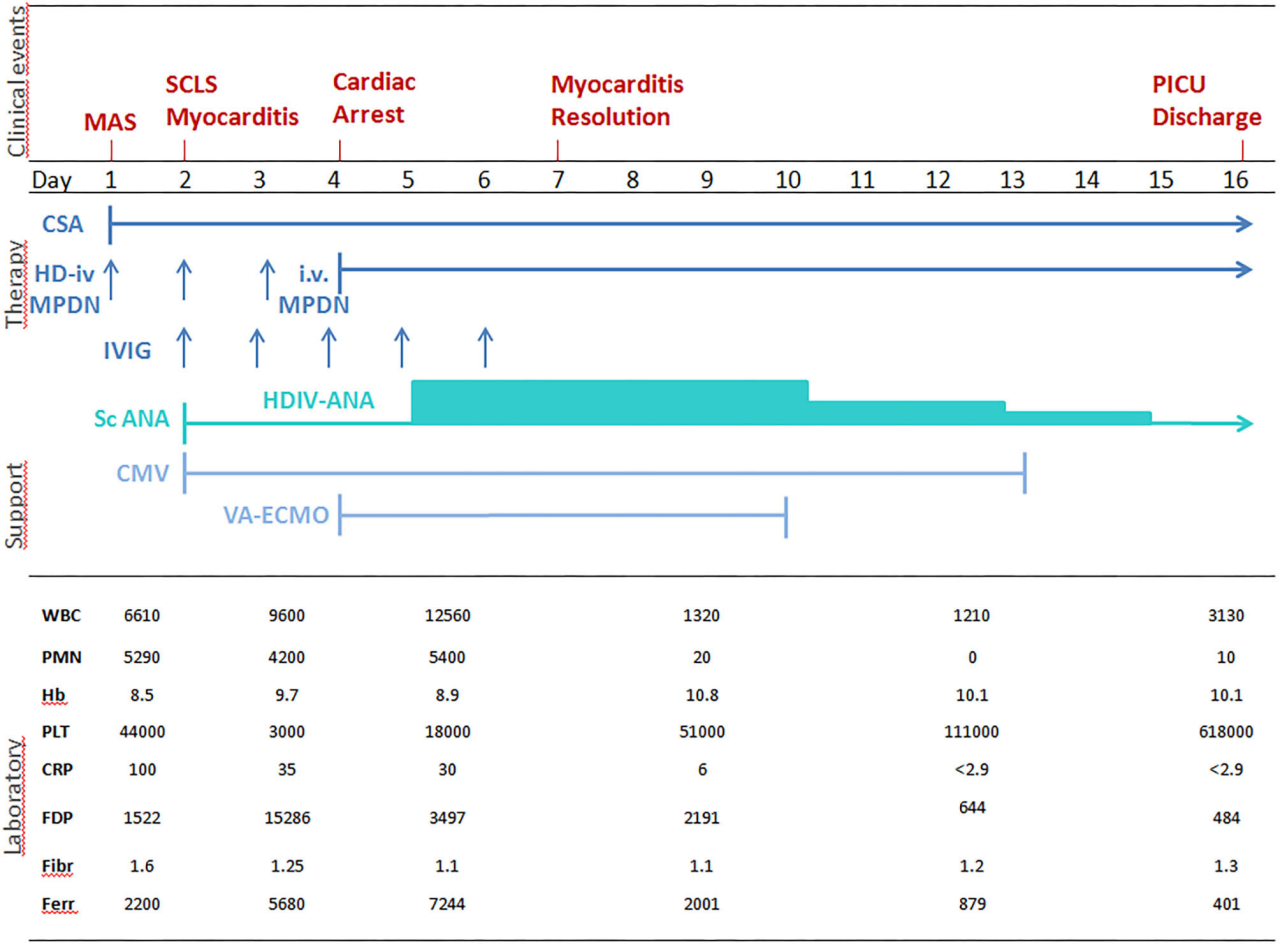
Correlation between clinical aspects, laboratory tests and treatment of sJIA-related MAS. WBC, white blood cells n./mm^3^; PMNs, polymorphonuclear cells n./mm^3^; Hb, hemoglobin gr/dl; PLT, platelets × 10^3^/mm^3^; CRP, C-reactive protein mg/l (n.v. <5); FDP, fibrinogen degradation products, μg/l (n.v. <250); FBG, serum fibrinogen g/l, (n.v.1.5 – 4.5); FER, ferritin μg/l (n.v. 20 – 250); IV HD-MPDN, intravenous methylprednisolone 30 mg/Kg/die iv; CSA, cyclosporine-A 2 mg/Kg/day; IVIG, intravenous immunoglobulin 400 mg/Kg/day; scANA, subcutaneous Anakinra 2 mg/Kg/day; HDIV-ANA, high dose intravenous Anakinra 2 mg/Kg/6 hours; CMV, Conventional mechanical ventilation; VA-ECMO, veno-arterial extracorporeal membrane oxygenation); SCLS, systemic capillary leak syndrome; MAS: macrophage activation syndrome.

**Figure 2:**
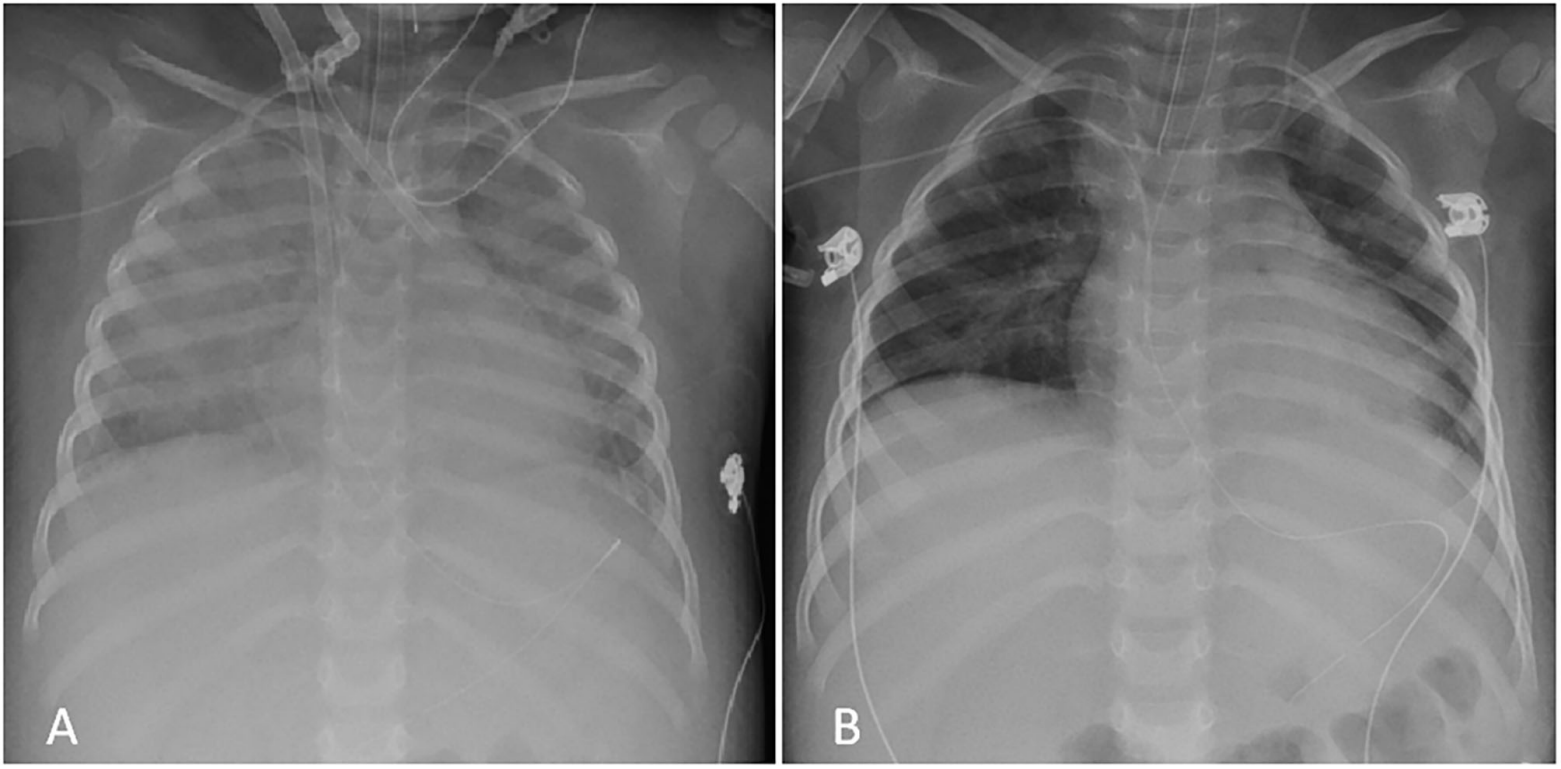
Chest X-Ray during PICU admission. **(A)** Severe bilateral interstitial thickening and right perihilar consolidation soon after VA-ECMO placement (day 4); **(B)** Complete resolution of the pattern after HDIV-ANA treatment at PICU discharge (day 16).

Laboratory tests showed: Hb 8.5 g/dl, PLT 44,000/mm3; FDP 1522 ug/L, fibrinogen 1.6 g/L, CRP 100 mg/L, AST 57 U/L, ALT 52 U/L; ferritin 2,200 ug/L; triglycerides 2.86 mmol/L. Suspecting an incipient MAS, high doses IV MDPN and oral Cyclosporin A (CSA, 2 mg/Kg/day) were started (Figure 1). 24 h later (day 2) he presented a systemic capillary leak syndrome (SCLS) with rapidly increasing weight (+ 5 Kg, ~30% BW), persistent hypotension (85/45 mmHg), tachycardia (160/min) and oliguria. He was urgently admitted into the Pediatric Intensive Care Unit (PICU) where inotropic and vasoactive support was maximized and conventional mechanical ventilation soon after started. Echocardiography revealed significantly increased myocardial thickness and echoic appearance, consistent with acute myocarditis. As summarized in Figure 1, IV Immunoglobulin (IVIG 400 mg/Kg/day for 5 days) and subcutaneous ANA (2 mg/Kg/day) were added. Indeed, multiple blood and platelet transfusions were needed to treat severe anemia and thrombocytopenia. Unfortunately, 48 h later (day 4) an episode of acute hypotension, bradycardia and oxygen desaturation led to cardiac arrest needing cardiopulmonary resuscitation and then VA-ECMO because cardiac function did not improve (Figure 2). On hemodynamic stability, upon informed consent by the parents, we started high dose intravenous ANA treatment (HDIV-ANA, 2 mg/Kg q6h). Starting from 72 h later (day 7), serial echocardiography revealed progressive resolution of myocarditis therefore VA-ECMO support was gradually decreased and definitively weaned off on day 10. Laboratory test showed improvement of MAS markers (Figure 1) but also the presence of a severe neutropenia (PMNs 0–100/mm3). Bone marrow aspirate confirmed poor PMNs representation, mainly consisting of pro-myelocytes with rare residual aspects of hemophagocytosis. Suspecting iatrogenic neutropenia, HDIV-ANA was gradually reduced to the maintenance subcutaneous dose of 2 mg/kg/day without resolution of neutropenia. Therefore, on day 22, ANA was stopped and neutropenia gradually resolved. The patient was discharged from the PICU 16 days after admission (Figure 2). Genetic analysis for familial hemophagocytic lymphohistiocytosis revealed a mutation in the PRF1 gene [c.(272C>T) p.(Ala91Val)] in heterozygosis, reported in sJIA-related MAS (11). 49 days after admission the patient was discharged on oral PDN (1 mg/Kg/day) and CSA (2 mg/Kg/day). Neither neurological signs or symptoms nor other internal organ consequences related to MAS were reported. Interestingly, a few months later, on tapering down of therapy, he relapsed with fever and increased ferritin and CRP. Subcutaneous ANA (2 mg/Kg/day) was restarted with rapid clinical and laboratory improvement and no side effects. During the follow up visits, corticosteroid and CSA therapy were gradually tapered down until stopping 4 and 6 months after discharge, respectively. Indeed, since parents reported difficulty maintaining adherence to ANA daily injections, 6 months after discharge, we switched to Canakinumab, a long-acting human monoclonal antibody targeting IL-1β, at the dose of 4 mg/Kg q4wk and then gradually tapered down. Currently, after 24 months, the disease is in clinical remission on medication (Canakinumab 4 mg/Kg q6wk).

## Discussion

MAS is a life-threatening condition, most commonly reported as a complication of sJIA and triggered by infections in up to one-third of the patients (2). It is the result of a cytokine storm that lead to a dysregulated inflammatory activation of the immune system, with rapid progression to multiorgan failure if not promptly diagnosed and properly treated. Early diagnosis is still a clinical challenge since there is no diagnostic test able to differentiate MAS from a flare of the underlying systemic inflammatory disease (12). In 2016, Classification Criteria of MAS in sJIA were proposed providing a sensitive and specific tool for the recognition of MAS in this subset of patients (13). The main clinical and laboratory features of MAS include unremitting high-degree fever, hyperferritinemia, pancytopenia, fibrinolytic consumptive coagulopathy and liver dysfunction (12). The goal of therapy is controlling and, possibly, stopping the immune hyper-activation and cytokine related overproduction as quickly as possible. Treatment usually includes high dose corticosteroids and immunosuppressive agents in refractory cases. Recently, the better understanding of the disease pathogenesis, in particular the crucial role of IL-1, suggested adopting a more targeted approach based upon the use of selective cytokine inhibitors. Although no standardized guidelines are available to date, the use of ANA, a recombinant IL-1 receptor antagonist, has been reported in adult-onset Still Disease, SLE and Undifferentiated Connective Tissue Disease (UCTD) complicated by MAS, sometimes with cardiac involvement (7, 8, 14). In pediatric MAS, treatment with ANA has been also proposed, pointing out the need for a higher doses regimen in severe refractory cases (3–5, 15). Although supportive evidence is still limited, intravenous ANA has also been used in patients with cytokine storm syndromes (5, 14) probably because it enables higher and faster maximal plasma concentration as compared with subcutaneous administration (5, 16). Moreover, pharmacokinetic studies have shown an increasing ANA half-life according with Body Mass Index, if administered subcutaneously, meaning that patients with greater adipose tissue have slower drug's transport and absorption (5, 16). Therefore, it is reasonable that in patients with subcutaneous edema or anasarca the intravenous route might be preferable.

In our case, the intravenous ANA administration was a forced choice, partly due to the prominent generalized edema related to the SCLS and partly to the severe risk of bleeding, related both to MAS and ECMO. We now recognize that this choice has been successful. Along with the unexpected favorable result, no major adverse events were noticed, except for a transient neutropenia, already reported (17).

Based on our experience, HDIV-ANA is a safe and effective treatment for refractory life-threatening sJIA-related MAS, even if complicated by acute fulminant myocarditis. Based on our positive experience, this therapeutic approach may be also considered in the current pandemic COVID-19 emergency where myocardial injury has been recently reported and where recent evidence showed interleukin-β-driven MAS-like complication, triggered by SARS-CoV-2 virus, as predictor of bad outcome (18–20).

## Data Availability Statement

The original contributions presented in the study are included in the article/supplementary material, further inquiries can be directed to the corresponding author/s.

## Author Contributions

AM, GM, and FZ decided the personalized treatment, reviewed the literature about Anakinra use in MAS and received final approval by the hospital pharmacy, and general management department for its use. AM collected all data, conceptualized, and wrote the manuscript. FZ supervised the study team and critically reviewed the manuscript for important intellectual aspect of the work. All authors approved the final manuscript as submitted and agreed to be accountable for all aspects of the work. All authors took care of the patient during hospitalization, reviewed, and approved the final version of the manuscript.

## Conflict of Interest

The authors declare that the research was conducted in the absence of any commercial or financial relationships that could be construed as a potential conflict of interest.

## Abbreviations

ALT: alanine aminotransferase
ANA: anakinra
AST: aspartate aminotransferase
CRP: C-reactive protein
CSA: cyclosporin A
ESR: erythrocyte sedimentation rate
FDP: fibronogen degradation products
Hb: hemoglobin
HDIV-ANA: high dose intravenous anakinra
IV: intravenous
IVIG: intravenous immunoglobulin
MAS: macrophage activation syndrome
PDN: prednisone
PICU: pediatric intensive care unit
PLT: platelets
PMNs: polymorphonuclear leukocytes
SCLS: systemic capillary leak syndrome
sJIA: systemic juvenile idiopathic arthritis
VA-ECMO: veno-arterial extracorporeal membrane oxygenation
WBC: white blood cells.
